# Evaluation of safety and anti-obesity effects of DWP16001 in naturally obese dogs

**DOI:** 10.1186/s12917-022-03324-2

**Published:** 2022-06-22

**Authors:** Beomseok Rhee, Rahman Md Mahbubur, Changfan Jin, Ji-Soo Choi, Hyun-Woo Lim, Wan Huh, Joon Seok Park, Jumi Han, Sokho Kim, Youngwon Lee, Jinho Park

**Affiliations:** 1 KNOTUS Co., Ltd., Research Center, Incheon, Republic of Korea; 2 Department of Veterinary Medical Imaging, College of Veterinary Medicine, Chungnam National University, Daejeon, Republic of Korea; 3 Daewoong Pharmaceutical Co., Ltd., Yongin, Republic of Korea; 4 Department of Veterinary Internal Medicine, College of Veterinary Medicine, Jeonbuk National University, Iksan, Republic of Korea

**Keywords:** Dogs, DWP16001, Obesity, SGLT2 inhibitor

## Abstract

**Background:**

The aim of this study was to investigate the anti-obesity effects of DWP16001, a sodium-glucose cotransporter-2 (SGLT2 inhibitor), in naturally obese dogs. A total of 20 dogs were divided into four equal groups: one obese control (OC group), and three treated groups; DWP0.2 group, DWP0.5 group, and DWP1 group. OC group fed with food for maintenance and treated groups were fed with food for maintenance with 0.2 mg/kg DWP16001, 0.5 mg/kg DWP16001 and 1 mg/kg DWP16001, respectively. The food for maintenance was provided to dogs as 2 RER (Resting energy requirement) in kcal and DWP16001-supplemented food was administered once a day for 8 weeks.

**Results:**

Body condition score, body weight, and fat thickness were significantly reduced (*P* < 0.05) in the DWP0.2 group compared with the OC group, respectively without affecting the food consumption. At the 10^th^ week the food consumption rate was 101.35 ± 2.56, 166.59 ± 4.72, 98.47 ± 1.44 and 123.15 ± 2.45% compared with initial food consumption rate. Body fat percentage, chest and waist circumference, blood glucose, and insulin were reduced compared to OC group but not significantly different from those of the OC group during experimental period. Serum alanine aminotransferase, alkaline phosphatase, creatine phosphokinase, and creatinine were significantly reduced in DWP0.2 group on 8 weeks. Serum cholesterol and triglycerides were reduced but not significantly. No specific adverse effects were observed throughout the experiment, and hematological parameters were unchanged. The results indicate that DWP16001 was not harmful to the dogs in our study and might have anti-obesity effects in naturally obese dogs.

**Conclusions:**

The above results and discussion suggest that DWP16001 is safe and might have anti-obesity effects in naturally obese dogs.

## Background

Obesity contributes to the morbidity of many diseases, including diabetes, dyslipidemia, cardiovascular disease, neurological diseases, gall bladder disease, musculoskeletal disorders, urinary tract and reproductive disorders, and certain types of cancer [1, 2]. Obesity results from higher energy intake than expenditure, which can lead to dysfunction of adipocytes, triggering pathological disorders of multiple organs and systems [2]. Worldwide, obesity and overweight disorder are reported to affect 609 million and 1.9 billion adults, respectively, in each year, representing about 39% of the global population [3].

The large body of literature addressing obesity and its prevention and treatment indicates the importance of obesity in human medical science [3]. Last decade, one health which the collaborative efforts of multiple disciplines working locally, nationally, and globally for people, animals and environment is attracting attention [4]. Many studies and opinions of obesity in companion animal suggested relationship between human obesity and dog obesity [5, 6]. There are major risk factors for obesity, such as feeding habits, physical inactivity, and genetic are similar among humans and dogs [7, 8]. In addition, the lifestyles of companion animal are almost fully owner-dependent; consequently, the attitudes, behaviors, and habits of their owners, such as offering excessive foods and not allowing for optimum exercise, and life events such as sterilization, can contribute to owner-influenced canine obesity [9, 10]. The human-animal bond is a predominant factor in the association between obesity in people and their dogs.

The prevalence of overweight and obesity in dogs is similar to that of humans. The prevalence of canine overweight and obesity in Brazil is 40.5% [10], although similar study in USA reported that overweight and obesity range from 19.7–59.3% in companion dogs [11]. It has been demonstrated that obesity induces similar comorbidities in companion animals, such as metabolic abnormalities, endocrinopathies, orthopedic disorders, cardiorespiratory disease, urogenital disorders, and neoplasia [12]. In the Labrador Retriever, moderate obesity has been reported to reduce life expectancy by almost two years [13]. Therefore, prevention of obesity in canine species should be taken seriously.

The sodium-glucose cotransporter-2 (SGLT2) inhibitor DWP16001 is a new drug for treatment of type 2 diabetes, developed by Daewoong Pharmaceutical Co., Ltd (Seoul, Republic of Korea), and is currently undergoing a Phase 3 clinical trial in Korea (Registration No. NCT04632862) after getting promising outcome at Phase 2 clinical trial (Registration No. NCT04014023) [14]. In the present study, the food of naturally obese dogs was supplemented with DWP16001 to evaluate the anti-obesity effects by assessing body condition score (BCS), chest and waist circumference, hematological parameters, and serum and urinary biochemical profiles.

## Results

### Effects of DWP16001 on BCS, body weight, body fat percentage, fat thickness, chest and waist circumference

**Fig. 1:**
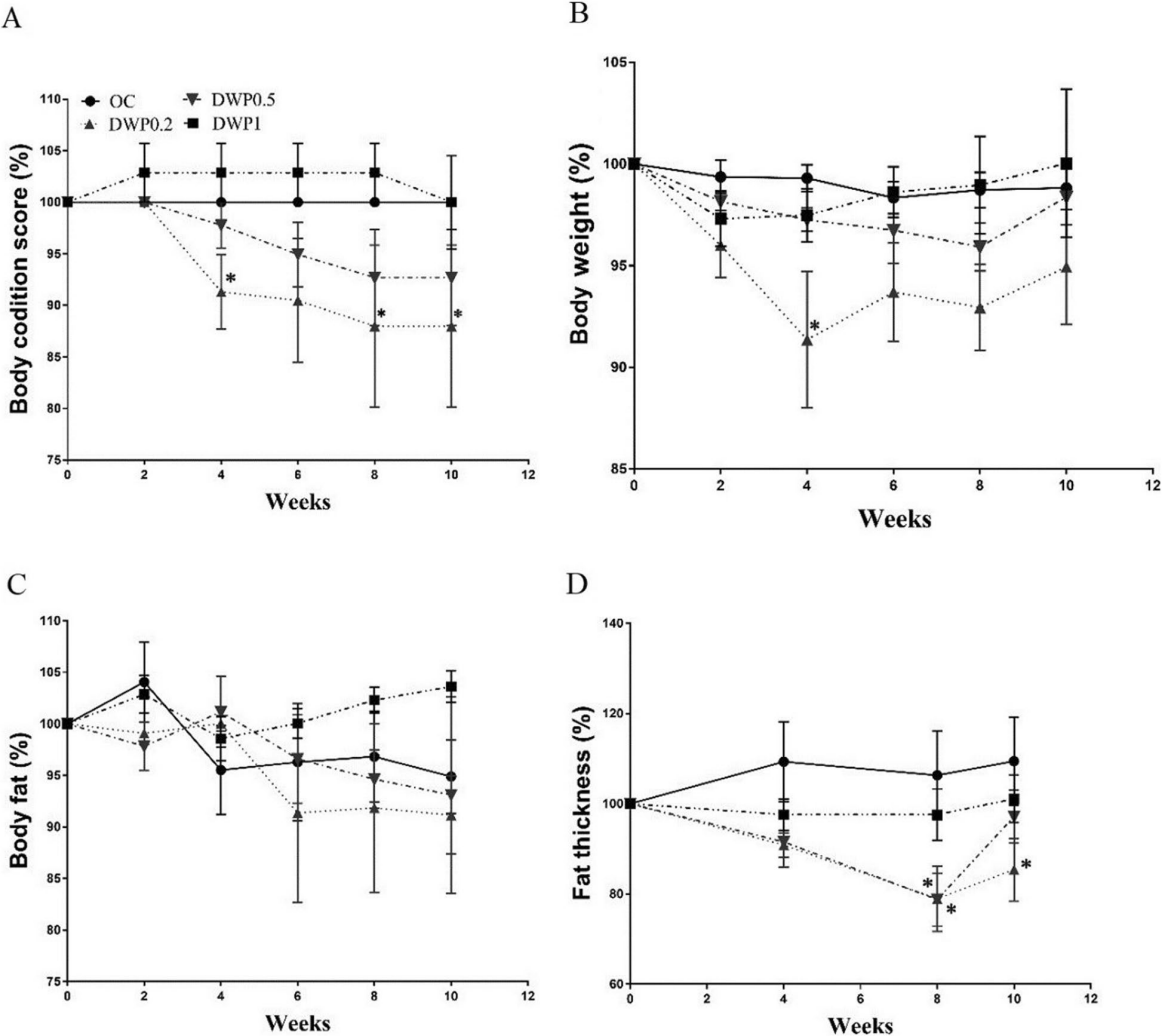
Effects of DWP16001 on changes of body condition score percentage, changes of body weight percentage, changes of body fat percentage and changes of fat thickness percentage of obese dogs. (**A**) Changes of body condition score percentage of obese dogs, (**B**) Changes of body weight percentage of obese dogs, (**C**) Changes of body fat percentage of obese dogs, (**D**) Changes of fat thickness percentage of obese dogs. OC: Obese control group; DWP0.2 group: DWP16001 0.2 mg/kg/day, DWP0.5 group: DWP16001 0.5 mg/kg/day, DWP1 group: DWP16001 1 mg/kg/day. Data are expressed as Mean ± S.D. * A significant difference at *p* < 0.05. *: *p* < 0.05, Bonferroni post hoc test following two-way ANOVA versus OC group

The BCS of the DWP 0.2 group was significantly lower (*p* < 0.05) than that in the OC group at 4, 8, and 10 weeks after the start of administration. A trend toward reduction of BCS was observed in the DWP 0.5 group over the entire experimental period. In contrast, the BCS of the DWP1 group was slightly increased but lower than that in the OC group by the end of the experiment (Fig. 1A). The BW of the DWP 0.2 group was significantly lower (*p* < 0.05) than that of the OC group at 4 weeks after the start of administration until the end of the trial, and the change of BW was lowest among all groups. Similar reductions of BW occurred in the DWP 0.5 group: BW was reduced at 2 and 4 weeks and then slowly increased (Fig. 1B). There was no significant difference in body fat percentage among all the groups, but it was lowest in the DWP 0.2 group (Fig. 1C). Reduced fat thickness was measured in all treated groups, but this change was significant only at 8 and 10 weeks in the DWP 0.2 group (Fig. 1D). At the point of 8 weeks, the chest and waist circumference of all three treated groups were reduced compared to initial, but the reductions were not significant at any time point (Table 1).

**Table 1:** Change rates of chest and waist circumferences (%)

**Weeks**	**OC**	**DWP0.2**	**DWP0.5**	**DWP1**
Change rates of chest circumferences (%)				
0	100.00 ± 0.00	100.00 ± 0.00	100.00 ± 0.00	100.00 ± 0.00
2	99.39 ± 3.13	99.20 ± 3.44	98.19 ± 4.31	94.79 ± 6.67
4	99.64 ± 3.44	97.16 ± 2.74	97.16 ± 3.23	99.81 ± 2.43
6	96.92 ± 5.74	95.08 ± 3.96	95.60 ± 4.55	100.54 ± 4.98
8	96.99 ± 4.44	96.08 ± 2.60	93.90 ± 3.11	99.20 ± 4.49
10	99.52 ± 6.24	99.18 ± 3.01	96.43 ± 2.76	100.24 ± 5.15
Change rates of waist circumferences (%)				
0	100.00 ± 0.00	100.00 ± 0.00	100.00 ± 0.00	100.00 ± 0.00
2	101.93 ± 8.89	100.53 ± 4.71	99.57 ± 1.71	95.97 ± 11.67
4	104.05 ± 6.64	97.39 ± 12.15	98.45 ± 4.94	99.18 ± 8.09
6	96.08 ± 7.08	98.96 ± 15.47	96.30 ± 3.83	103.75 ± 10.54
8	102.61 ± 6.40	98.89 ± 11.98	93.84 ± 5.54	99.76 ± 12.53
10	103.54 ± 2.93	101.98 ± 10.78	96.00 ± 4.72	101.33 ± 14.44

Data are expressed as Mean ± S.D

*OC* Obese normal control, *DWP0.1* DWP16001 0.2 mg/kg/day, *DWP0.2* DWP16001 0.5 mg/kg/day, *DWP1* DWP16001 1 mg/kg/day

^*^A significant difference at *p* < 0.05 level compared to the OC

### Rate of change in food consumption and resting energy requirement

At the 10th week the food consumption rate was 101.35 ± 2.56, 166.59 ± 4.72, 98.47 ± 1.44 and 123.15 ± 2.45% compared with initial food consumption rate. Interestingly, food consumption increased significantly (*p* < 0.001) in the DWP 0.2 and DWP1 groups compared with the OC group but decreased in the DWP 0.5 group over the 10-week test period (Table 2). Analysis of resting energy requirement (RER, *i.e.*, base level of calories required) at 4 and 8 weeks after the start of administration of DWP16001 showed that the RER of the DWP 0.2 group was significantly lower than that in the OC group (*p* < 0.05; Table 2).

**Table 2:** Change rates of food consumption and resting energy requirement (%)

**Weeks**	**OC**	**DWP0.2**	**DWP0.5**	**DWP1**
Change rates of food consumption (%)				
0	100.00 ± 0.00	100.00 ± 0.00	100.00 ± 0.00	100.00 ± 0.00
2	102.10 ± 1.31	126.52 ± 5.48***	95.13 ± 1.67***	100.80 ± 4.20
4	101.38 ± 1.25	146.64 ± 5.37***	92.34 ± 2.10***	116.02 ± 3.09***
6	99.75 ± 1.19	153.71 ± 4.11***	92.30 ± 1.60***	121.84 ± 2.50***
8	98.74 ± 1.41	168.32 ± 4.80***	93.64 ± 1.67*	121.88 ± 2.36***
10	101.35 ± 2.56	166.59 ± 4.72***	98.47 ± 1.44	123.15 ± 2.45***
Change rates of resting energy requirement				
0	100.00 ± 0.00	100.00 ± 0.00	100.00 ± 0.00	100.00 ± 0.00
2	99.51 ± 1.39	96.98 ± 2.69	98.63 ± 0.72	97.75 ± 2.32
4	99.48 ± 1.10	93.41 ± 5.79	97.93 ± 1.00	98.11 ± 2.18
6	98.74 ± 1.32	95.21 ± 4.10	97.54 ± 2.73	98.95 ± 2.07
8	99.04 ± 1.45	94.64 ± 3.59*	96.92 ± 2.00	99.19 ± 4.05
10	99.12 ± 1.77	96.15 ± 4.75	98.79 ± 2.30	99.97 ± 6.15

Data are expressed as Mean ± S.D

*OC* Obese normal control, *DWP0.1* DWP16001 0.2 mg/kg/day, *DWP0.2* DWP16001 0.5 mg/kg/day, *DWP1* DWP16001 1 mg/kg/day

^*^A significant difference at *p* < 0.05 level compared to the OC

**A significant difference at *p*<0.01 level compared to the OC

***A significant difference at *p*<0.001 level compared to the OC

### Effects of *DWP16001* on hematological and serum biochemical profiles

There were no significant differences in hematological findings among the treatment groups over the 10-week test period, except for PLT. The PLT count of the DWP1 group was significantly lower than that in the OC group (*p* < 0.05; Table 3). Serum TC and TG concentrations in the DWP0.2 group were lower than those in the OC group. Fasting glucose and serum insulin concentrations of all treated groups were reduced compared with those in the OC group, but only GLU in the DWP1 group and insulin concentration in the DWP0.5 group were significantly lower than those in the OC group. Serum TG concentration of treated groups (DWP0.2, 142.00 ± 95.55 mg/dL; DWP0.5, 133.86 ± 70.19 mg/dL; DWP1, 282.60 ± 419.03 mg/dL) was lower than that in the OC group (361.60 ± 622.34 mg/dL). Furthermore, serum GGT, ALT, ALP, CPK, and CRP concentrations declined significantly in the DWP0.2 group compared with the OC group. Urinary GLU excretion was greater in treated groups, especially in the DWP0.5 and DWP1 group (Table 3). Indeed, the glucose excretion level was increased in time dependent after the start of the test substance administration in DWP16001 treated group until 8 weeks (data not shown).

**Table 3:** Effects of DWP16001 on blood, serum and urinary biochemistry of obese dogs in 8 weeks

	**OC**	**DWP0.2**	**DWP0.5**	**DWP1**
WBC (× 10^3^μL)	7.93 ± 2.83	11.12 ± 3.69	7.75 ± 2.04	10.01 ± 3.35
RBC × 10^6^μL)	6.90 ± 0.99	6.92 ± 0.79	72.22 ± 0.32	6.55 ± 1.08
Hb (g/dL)	16.36 ± 1.98	16.34 ± 2.68	16.40 ± 0.70	16.16 ± 2.15
Hct (%)	47.94 ± 7.61	49.86 ± 7.08	50.26 ± 1.94	46.76 ± 6.13
Platelet (× 10^3^/μL)	422.60 ± 12.98	392.20 ± 11.16	307.40 ± 6.02	299.10 ± 7.65*
Glu (mg/dL)	113.80 ± 10.85	107.20 ± 10.85	97.77 ± 10.19	94.00 ± 13.47
Insulin (ng/mL)	133.27 ± 16.43	126.60 ± 15.45	99.82 ± 18.48*	110.75 ± 21.96
TC (mg/dL)	236.40 ± 69.21	183.84 ± 36.01	298.00 ± 71.14	309.00 ± 129.40
TG (mg/dL)	361.60 ± 622.34	142.00 ± 95.55	133.86 ± 70.19	282.60 ± 419.03
TP (mg/dL)	6.48 ± 0.66	6.78 ± 0.66	6.43 ± 0.32	6.14 ± 0.54
ALB (g/dL)	3.04 ± 0.30	3.48 ± 0.42	2.95 ± 0.19	2.92 ± 0.29
GGT (IU/L)	13.20 ± 14.55	7.80 ± 1.64	9.46 ± 5.37	21.60 ± 29.36
ALT(IU/L)	50.60 ± 29.33	18.84 ± 4.00*	36.80 ± 14.43*	38.60 ± 19.72
AST(IU/L)	44.80 ± 16.21	31.00 ± 1.83	31.84 ± 12.92	110.00 ± 167.92
ALP(IU/L)	211.60 ± 199.74	84.60 ± 63.96*	146.20 ± 121.67	186.20 ± 184.59
CPK(IU/L)	129.40 ± 35.82	111.12 ± 30.97*	136.50 ± 42.99	260.40 ± 126.79
BUN (mg/dL)	15.12 ± 6.30	18.70 ± 10.29	22.25 ± 8.64	23.44 ± 13.17
CRE (mg/dL)	1.20 ± 0.31	0.78 ± 0.21*	0.82 ± 0.15	0.92 ± 0.36
Urinary GLU (grade)	0	2 ± 0.12	4	4

*OC* Obese control group, *DWP0.2 group* DWP16001 0.2 mg/kg/day, *DWP0.5 group* DWP16001 0.5 mg/kg/day, *DWP1 group* DWP16001 1 mg/kg/day

Data are expressed as Mean ± S.D

^*^A significant difference at *p* < 0.05, post hoc test following one‐way ANOVA versus OC

*RBC* Red blood cell, *Hct* Hematocrit, *Hb* Hemoglobin, *WBC* White blood cell, *PLT* Platelets, *AST* Aspartate aminotransferase, *ALT* Alanine aminotransferase, *ALP* Alkaline phosphatase, *CPK* Creatine phosphokinase, *TP* Total protein, *GLU* Glucose, *TC* Total cholesterol, *TG* Triglyceride, *ALB* Albumin, *GGT* Gamma-glutamyl transferase, *BUN* Blood urea nitrogen and *CRE* Creatinine

### Adverse reactions

During Week 2 of the test, one dog in the DWP0.2 group developed hematuria, and one dog in the DWP1 group developed conjunctivitis with dry eye, both of which improved without treatment. Compared with other agents, adverse effects of DWP16001 were not observed including diarrhea, flatulence, bloating, abdominal pain, and dyspepsia.

## Discussion

Treatment of type 2 diabetes and obesity with SGLT2 inhibitors has shown promise, as these drugs reduce hyperglycemia and body weight gain in mice [15]. Human clinical trials also proved that SGLT2 inhibitors reduce body weight about 1.5–2 kg compared to placebo group [16]. SGLT2 is important in renal glucose reabsorption; selective blockade of SGLT2 regulates blood glucose to appropriate levels by promoting urinary excretion of glucose. SGLT2 inhibitors act in an insulin-independent manner and are not associated with pancreatic b-cell function. Therefore, SGLT2 inhibitors are attracting attention as alternative or combination therapies for type 2 diabetes. Several SGLT2 inhibitors, including empagliflozin, dapagliflozin, canagliflozin, and ipragliflozin, have been approved for type 2 diabetes [15]. Recently, the SGLT2 inhibitor DWP16001 was shown to be distributed to the kidney (target organ) and to exert more selective and sustained SGLT2 inhibition compared to other SGLT-2 inhibitors such as ipragliflozin and dapagliflozin [17]. In this study, we investigated the anti-obesity effects when the dogs are fed food for maintenance plus supplemented DWP16001 and safety of DWP16001 in naturally obese dogs. The results of the study demonstrated that DWP16001 reduces BCS, body weight, body fat percentage, fat thickness, and chest and waist circumference without affecting food consumption. The most effective results were found when food was supplemented with 0.2 mg/kg DWP16001.

The DWP16001-treated groups in our study had reduced blood glucose concentration and increased urinary glucose excretion. These results are consistent with those of many previous clinical studies that have reported that orally administered SGLT2 inhibitors reduce body weight and improve hyperglycemia and T2D through induction of urinary glucose excretion [18–20]. Furthermore, SGLT2 inhibitors have been shown to induce reductions in body weight and fat mass in mouse models fed a high-salt and high-fat diet [15, 21]. In obese subjects, elevated serum TC, TG, AST, ALT, CPK, BUN, and CRE have been reported [15, 22] all of which were showed reduction in DWP16001 treated group, although it cannot be definitively concluded due to the difference in the type of food fed, it is thought that the DWP16001 has had some influence. Increasing TC and TG concentrations are indicators of hyperlipidemia [2, 22] which were increased in OC group and lowered by DWP16001 treatment indicating anti-lipidemic effect [23]. Elevated BUN and CRE concentrations are seen in kidney disorders, and increased AST, ALT, and CPK concentrations are indicators of fatty liver which were increased in OC group and lowered by DWP16001. Our findings suggest DWP16001 treatment might have effects on obesity-induced pathological disorders.

Indeed, BCS assessment is widely used to identify obesity in pet animals, with a nine-stage scoring system typically being recommended. Under the nine-stage scoring, dogs with a BCS of 6 or 7 are considered overweight, whereas those with a BCS of 8 or 9 are obese [10, 24]. This study comprised dogs with BCSs of 7 or more, and the results indicated that DWP16001 treatment significantly reduced the BCS after four weeks of administration in the DWP 0.2 group. Similarly, body weight, body fat percentage, fat thickness, and chest and waist circumference were reduced. The reductions of body fat percentage and chest and waist circumference might have been secondary to reduction of fat thickness in this study. Moreover, the decrease in glucose (and hence calories available) induced by DWP16001 might have induced increased lipid mobilization, resulting in reduction of fat mass and body weight [15]. A common side effect of medication-related weight loss is long-term loss of appetite [25, 26]. Interestingly, food consumption in the DWP 0.2 and DWP1 groups increased significantly despite reductions in BCS and BW but was reduced in the DWP 0.5 group. These findings indicate that the appropriate dose of DWP16001 could reduce body weight gain and obesity without altering feeding behavior. Urinary glucose excretion results in physiological compensatory sugar intake [27]. As a result of this test, no effect on consistent feed intake was observed at all doses, but an increase in feed intake was observed in the DWP0.2 group might be for compensatory glucose intake whose weight loss effect was confirmed. Based on this result, excellent weight loss effect despite an increase in feed intake in the DWP0.2 group was observed. Likewise, it has been reported that empagliflozin, another SGLT2 inhibitor, increased food intake significantly despite reductions in body weight gain in mice [15, 27].

Thrombocytopenia or platelet dysfunction results in skin and mucosal bleeding, which are associated with hematuria [28]. In the present study, we also analyzed hematuria and thrombocytopenia for the experimental dogs. Platelet count was significantly reduced in the DWP1 group compared with the OC group, and hematuria was found in one dog in the DWP0.2 group, which recovered without treatment. Therefore, hematuria and thrombocytopenia do not appear to be correlated in this study. In the DWP1 group, one dog developed conjunctivitis with dry eye, which improved without treatment. Dry eye is strongly depended on environmental temperature, air flow and moisture contents [29]. The dogs here owned by owner was not possible to provide optimum environmental factors. The owner was suggested to change the environmental condition but it was not confirmed that this factor induced dry eye. Additionally, dry eye is associated with hyperglycemia and hyperlipidemia [30], but these conditions, where present in the study dogs, were not severe. Although SGLT2 inhibitors can induce dry skin, dermatitis, and subcutaneous tissue including rash, eruption, urticaria, erythema, and eczema [31], there have been no reports of dry eye induction. Continuous consumption of anti-obesity drugs can induce other adverse effects, including diarrhea, bloating, abdominal pain, and dyspepsia [26]. While such symptoms were not observed in our study, additional experimental studies are necessary to establish the long-term efficacy and safety of DWP16001.

## Conclusions

The above results and discussion suggest that DWP16001 is safe and might have anti-obesity effects in naturally obese dogs. Additionally, the DWP16001 0.2 and 0.5 mg/kg/day groups showed a trend toward reduced body weight, body condition score, basic calorie requirement, chest and waist circumference, body fat percentage, and fat thickness, although it cannot be definitively concluded due to the difference in the type of food fed. In this study, DWP16001 was administered to naturally obese dogs in clinical practice. Thus, the owner and veterinarian evaluating each dog differed, the dogs were of different breeds and ages, and the number of individuals in each group was small. The results are considered to be the effect of administration of the DWP16001; however, further studies are needed to confirm the effects, as we did not find clear dose dependence of effects in our study population.

## Materials and methods

### Animal selection, grouping, and monitoring

Naturally obese but otherwise healthy adult dogs, regardless of breed, sex, and altering a dog’s reproductive status, were selected for this clinical trial. The dog owners provided informed consent for their pet to participate in the clinical trial from veterinary hospital. All dogs were fed with their ordinary food from owner and report food type, consumption quantity to veterinary hospital. All dogs were diagnosed with obesity according the findings of a physical examination, including body weight and body conditions score (BCS) of 7 or more, 9 stages in total [32]. Only one dog was analyzed per household to ensure individual feeding. The selected dogs were generally healthy without underlying disease, such as abnormalities of the liver, heart, and/or kidneys. Dogs used for breeding purposes or treated with long-acting steroids, drugs that affect endocrine conditions (such as lipid improvement, cholesterol inhibitors, diabetes drugs), or drugs that affect weight or energy consumption (such as phenobarbital) within 30 days prior to commencement of our trial were excluded. A total of 20 dogs were divided in four groups of 5 animals each randomly: one obese control (OC group), and three treated groups; DWP0.2 group, DWP0.5 group, and DWP1 group. OC group was fed with food for maintenance and three treated groups were fed with food for maintenance plus supplemented with 0.2 mg/kg DWP16001, 0.5 mg/kg DWP16001 and 1 mg/kg DWP16001 in the collagen capsule, respectively. The DWP16001 in the collagen capsule was administered once daily, and the food was also administered once daily for eight weeks. The breeds included in this study were Shetland Sheepdog (5/20), Maltese (3/20), Pompitz (2/20), Bichon Frise (1/20), Border Collie (1/20), Cocker Spaniel (1/20), Japanese Spitz (1/20), Labrador Retriever (1/20), Poodle (1/20), Shih Tzu (1/20), Wire Fox Terrier (1/20), Yorkshire Terrier (1/20), and mixed (1/20). In this study, 10/20 dogs were male (10/10 were neutered), and 10/20 dogs were female (7/10 were spayed). The age range was 3 to 11 years, and the average age of the dogs was 7.15 years. Nine of the 20 dogs were aged ≤ 6 years, while eight were 7–9 years of age, and three were ≥ 10 years of age. These dogs were divided in four groups of 5 animals each randomly for the experiments. The owners were asked to closely monitor their animals and requested to contact the investigators immediately if any abnormalities were found. In addition, each dog was examined by a veterinarian once weekly until the end of the trial.

All animal experiments were conducted in compliance with Animal Experimental Ethics Regulations noticed by the IACUC (Institutional Animal Care and Use Committee) in KNOTUS Co., Ltd. And this was approved by the IACUC in KNOTUS Co., Ltd. (Approval number: KNOTUS IACUC 20-KC-003). We complied with the ARRIVE (Animal Research: Reporting of In Vivo Experiments) guidelines.

### Measurement of body weight, chest and waist circumference, and resting energy requirement

Body weight (BW) and chest and waist circumference were measured just before administration of the first meal of DWP16001-supplemented food and once every two weeks thereafter for 10 weeks. The chest and waist circumference were measured at the thickest part of the chest and thinnest part between the chest and the hind legs, respectively, and the measurement was repeated four times to obtain a mean circumference. The resting energy requirement (RER) was calculated using the following equation from previous study [33, 34].

$$\mathrm{RER}\;\mathrm{in}\;\mathrm{kcal}/\mathrm{day}\:=\:70\;\mathrm x\;(\mathrm{initial}\;\mathrm{body}\;\mathrm{weights})^{0.75}$$

### Measurement of fat thickness

Fat thickness was assessed by measuring the right angle distance from the T10 spinal process to the skin using radiographs (Titan 2000 X-ray system, COMED Medical Systems Co., Ltd., Seoul, Korea) obtained on the first day of DWP16001 administration and at 4, 8, and 10 weeks thereafter.

### Hematology and serum and urine biochemical tests

Hematological testing was performed on the day before the first treatment and then once every two weeks thereafter for 10 weeks. Part of the blood collected on the test day was stored in a CBC tube containing EDTA-2 K, an anticoagulant, and the red blood cell (RBC) count, hematocrit (Hct), hemoglobin concentration (Hb), white blood cell (WBC) count, and platelet count (PLT) were assessed using an automatic blood analyzer (ADVIA 2120i™, Siemens Healthcare Diagnostics, Vienna, Austria), as described previously [35].

For biochemical analysis, part of the blood collected on the test day was transferred to a vacutainer tube containing a clot activator, left at room temperature for 15–20 min to solidify, and then centrifuged at 3000 rpm to obtain serum. Serum biochemical parameters were assessed using a Hitachi 7180 instrument (Hitachi, Tokyo, Japan) together with the scheduled hematological test [35]. The biochemical parameters assessed were aspartate aminotransferase (AST), alanine aminotransferase (ALT), alkaline phosphatase (ALP), creatine phosphokinase (CPK), total bilirubin (TBIL), glucose (GLU), total cholesterol (TC), triglycerides (TG), total protein (TP), albumin (ALB), gamma-glutamyl transpeptidase (GGT), blood urea nitrogen (BUN), creatinine (CRE), inorganic phosphorus (IP), calcium (Ca), and C-reactive protein (CRP). Serum insulin concentration was measured at 2, 4, 6, 8, and 10 weeks with a Quantikine Canine Insulin ELISA Kit (Catalog Number: DINS00, R&D Systems, Minneapolis, MN, USA) according to the manufacturer’s protocol. Urine biochemical parameters were assessed using a urine test strip at 2, 4, 6, and 10 weeks after the start of administration of the DWP16001, while urinary glucose (GLU) excretion was measured using a URiSCAN® Strip (YD Diagnostics CORP., Yongin-Si, Republic of Korea). Strip guided grade (0 ~ 4) revealed like as 0: Negative, 1: NA, 2: ≥ 250, 3: ≥ 500, 4: ≥ 1,000.

### Statistical analysis

Prism 5.03 software (GraphPad Software Inc., San Diego, CA, USA) was used for statistical analysis of the data. The result value compared with 100% was normalized based on the value before treatment with the test substance. Results are expressed as mean ± S.D. Data normality was assumed for the results of this test, and significance was tested between test groups using parametric one-way ANOVA. If significance was recognized, Dunnett's multiple comparison test was used to post-test was carried out. The level of significance was set at *p* < 0.05.

## Data Availability

The datasets supporting the conclusions of this article are included within the article.
